# Assessing nature exposure: a study on the reliability and validity of a Portuguese version of the nature exposure scale

**DOI:** 10.3389/fpsyg.2024.1290066

**Published:** 2024-03-04

**Authors:** Ana Picanço, Ana Moura Arroz, Noelline Tsafack, Ana Fuentes Sánchez, Enésima Pereira, Marta De Benedictis, Isabel R. Amorim, Maria da Luz Melo, Rosalina Gabriel

**Affiliations:** ^1^cE3c/GBA–Centre for Ecology, Evolution and Environmental Changes and CHANGE–Global Change and Sustainability Institute/Azorean Biodiversity Group and University of the Azores, Azores, Portugal; ^2^Avignon Université, Aix Marseille Université, CNRS, IRD, IMBE, Avignon, France; ^3^Núcleo de Investigação e Desenvolvimento em e-Saúde, Escola Superior de Saúde da UAc, Azores, Portugal

**Keywords:** nature exposure, psychometric tool, reliability, validity, connectedness to nature, subjective well-being, principal component analysis, confirmatory factor analysis

## Abstract

**Introduction:**

Engaging with nature has been widely acknowledged for its positive impact on well-being. Traditionally, assessments of nature exposure focus on estimating green space exposure and outdoor time. However, the Nature Exposure Scale (NES) offers a unique approach by evaluating the quality of nature experiences, encompassing both deliberate and spontaneous encounters.

**Methods:**

This study aimed to assess the validity and reliability of the NES in the context of Portugal. Exploratory Factor Analysis (PCA) and Confirmatory Factor Analysis (CFA) were employed to examine the underlying structure of the scale. Additionally, reliability assessments, along with tests of convergent and divergent validity, were conducted. Data were collected from a sample of 558 adults and 241 adolescents between 2016 and 2018.

**Results:**

The findings revealed strong internal consistency of the NES, supported by acceptable correlation values and robust factor loadings within a unidimensional model. The scale effectively predicted variations in nature exposure across diverse professional activities.

**Discussion:**

In summary, the 4-item NES emerged as a reliable tool for assessing nature exposure in various settings, effectively bridging a gap in the Portuguese context. The scale demonstrated potential for cross-cultural research and was particularly adept at predicting nature exposure in different professional contexts. The results suggest that the NES can enhance our understanding of the impact of nature on well-being in diverse cultural settings.

**Conclusion:**

The study underscores the reliability of the NES in assessing nature exposure in Portugal, paving the way for further exploration in Portuguese-speaking regions. The scale holds promise for advancing research on the relationship between nature and well-being, contributing valuable insights across different cultural contexts.

## 1 Introduction

The impact of human exposure to nature has garnered significant attention within the realms of health and well-being research and policy. This heightened interest is particularly pronounced in urbanized nations such as the United Kingdom, United States of America, Australia, and Canada where its impacts on health shows a negative exponential relationship between nature dose and urbanization (Largo-Wight et al., 2011; Shanahan et al., 2016a; Cox et al., 2017; Rugel et al., 2017; Holland et al., 2021). The allure of nature’s benefits is substantiated by numerous systematic investigations (Russell et al., 2013; Kuo, 2015; De Keijzer et al., 2016; Shanahan et al., 2016b; Jo et al., 2019; Trøstrup et al., 2019; Ricciardi et al., 2022) and narrative reviews (Holland et al., 2021; Jimenez et al., 2021).

These benefits extend not only to those who frequently immerse themselves in natural environments (Mayer et al., 2009; Pasanen et al., 2018; Wood et al., 2019; Wyles et al., 2019) but also to those who merely glimpse nature through images or windows (Kuo, 2015; Wyles et al., 2019). The array of advantages encompasses diverse domains, including enhanced general health indicators (Shanahan et al., 2016a) and specific physical health benefits such as reduced fatigue, stress, and anxiety (Pasanen et al., 2018; Repke et al., 2018; Jo et al., 2019; Javelle and Raoult, 2021), bolstered mental health (Trøstrup et al., 2019), improved overall well-being (Russell et al., 2013; Pasanen et al., 2018), and a positive body image (Swami et al., 2016; Trøstrup et al., 2019; Baceviciene et al., 2021). Beneficial associations are also found with the cognitive development in childhood and cognitive function in adulthood (de Keijzer et al., 2016; Weeland et al., 2019; Ricciardi et al., 2022), expedited therapeutic processes (Yeager et al., 2018) and heightened physical activity (Duvall, 2013; Shanahan et al., 2016b; James et al., 2017). Moreover, Soga and Gaston (2023) emphasized several pro-biodiversity behavioral changes, such as purchasing ecofriendly products, reducing pesticide use in domestic gardens, and donating to conservation organizations, among other positive effects.Furthermore, the potency of these effects appears to correlate with the biodiversity of the natural environments visited (Wood et al., 2019; Wyles et al., 2019) and is more pronounced when the exposure is deliberate (DeVille et al., 2021), when attention is focused on the natural context (Pasanen et al., 2018), and when individuals form a meaningful connection with specific natural elements or the environment as a whole (Ives et al., 2017; Martin et al., 2020). Moreover, the benefits of nature exposure may extend to the environment itself, as it fosters pro-environmental attitudes and behaviors (Frantz and Mayer, 2014; Zelenski et al., 2015). This influence is evident in activities such as volunteerism in environmental organizations and the promotion of community environmental awareness, both among children (Wells and Evans, 2003) and adults (DeVille et al., 2021). Nonetheless, some studies posit that the effects of nature exposure on health and well-being are partially mediated by the degree of an individual’s connection with the natural world (Mayer et al., 2009; Martin et al., 2020).

Various theoretical frameworks, including Wilson’s (1984) biophilia hypothesis emphasizing an innate connection to nature, Ulrich’s (1991) Psycho-evolutionary Theory exploring evolutionary-based psychological responses to the environment, and Kaplan’s (1995) Attention Restoration Theory highlighting the cognitive benefits of nature exposure, collectively offer insights into how interactions with nature influence human well-being, cognition, and emotional states; these frameworks have been empirically reviewed by Berto (2014).

However, the fundamental question remains: what exactly constitutes “nature,” and what does exposure to nature entail? Defining nature has proven to be a challenging and elusive task (Holland et al., 2021). The terms “nature” or “natural environments” are often juxtaposed with human-made, gray, built environments. Yet, these terms encompass a wide spectrum, ranging from green, blue, or brown natural areas to specific elements like plants, animals, hills, mountains, deserts, beaches, marshlands, rivers, streams, lakes, ponds, and oceans within both urban and rural settings, with varying degrees of human intervention (ranging from pristine areas to meticulously manicured gardens). Moreover, the experience of nature can take various forms, contingent on factors such as the spatial scale of the natural environment, individuals’ proximity to natural elements, the sensory channels through which nature is perceived (visual, auditory, etc.), the activities undertaken, and the level of mindfulness achieved while in natural settings, among other variables (Frumkin et al., 2017). Furthermore, exposure to nature can encompass both direct contact and mediated experiences (through windows, photographs, videos, sound recordings, etc.) with isolated natural environments or specific elements (Keniger et al., 2013; Rygal and Swami, 2021).

Remarkably, there is currently no standardized method for quantifying nature contact or exposure, whether direct or mediated, nor is there consensus on how to define and operationalize these concepts in research (Holland et al., 2021; Jimenez et al., 2021). At both individual and population levels, the most prevalent metrics for assessing nature exposure involve quantifying surrounding greenness and measuring time spent in natural settings using remote-sensing techniques or self-report instruments (Sadeh et al., 2021).

Global vegetation greenness, for instance, can gage the intensity of exposure by evaluating the density of vegetation cover in a land area, as opposed to measuring exposure frequency and duration. This can be accomplished through remote sensing or global positioning systems tracking via GPS devices or GPS-enabled smartphones, and estimated using the Normalized Difference Vegetative Index (NDVI) (Holland et al., 2021). Alternative, though less precise, techniques to assess greenness exposure include appraisals of green quantity in drone-captured photographs or users’ subjective evaluations of vegetation cover density (Wells and Evans, 2003; Holland et al., 2021). Remote sensing, such as LiDAR, involves analyzing reflectance patterns and comparing absorption and reflection values of red and near-infrared (NIR) light. LiDAR and NDVI are the most commonly employed sensing-based metrics in epidemiological studies, albeit their accuracy varies depending on vegetation density and soil characteristics (James et al., 2017; Sadeh et al., 2021). Several studies have highlighted the limitations of NDVI (Giannico et al., 2022), prompting the proposal of new measures like the Natural Space Index (Reid et al., 2018) or the affirmation of the advantage of existing ones such as Linear Spectral Unmixing (LSU) (Sadeh et al., 2021). The imperative to augment and complement nature exposure evaluation with additional indicators, considering intrinsic attributes of the natural context (e.g., natural space form or quality; Rugel et al., 2017) and observer-related factors (e.g., “access” or “distance”; Ekkel and de Vries, 2017), is underlined.

Besides methods that gage exposure through 2D or 3D indicators of spatial or contextual characterization (Giannico et al., 2022), novel methodological strategies are emerging to characterize individual experiences in terms of time spent (frequency and duration) in nature (Largo-Wight et al., 2011; White et al., 2019; Soga et al., 2021). Spatial approaches to measure nature exposure, however, fall short in capturing variations in how individuals intentionally or unintentionally experience nature, perceive it through various senses (e.g., smell, touch), engage in different activities, pay varying levels of attention to their natural surroundings, and perceived impact from it. Furthermore, as revised by Giannico et al. (2022), 2D indicators of nature exposure prove inadequate in assessing the quality of nature, as this quality is influenced by factors such as latitude, slope, climate conditions and biodiversity. These indicators are unable to evaluate three-dimensional (3D) characteristics like volume, height, or biomass, which may be relevant to human health (Giannico et al., 2022). They exhibit limited cross-study reproducibility, mainly due to inconsistencies in land use measures (Frumkin et al., 2017). To address these nuanced aspects of human contact with nature, self-report instruments such as questionnaires and psychometric scales have been employed (Holland et al., 2021). Self-reported instruments offer a promising avenue for accurately measuring nature exposure experiences. Importantly, subjective experiences, rather than mere exposure to nature, significantly influence well-being, particularly in terms of attention restoration through exposure to restorative environments (Berto, 2005; Moll et al., 2022). While a handful of self-reported questionnaires have been employed to measure nature exposure (Largo-Wight et al., 2011; Kamitsis and Francis, 2013; Swami et al., 2016; Wood et al., 2019; Baceviciene et al., 2021; Javelle and Raoult, 2021), only the Nature Contact Questionnaire (NCQ) (Largo-Wight et al., 2011) and the Nature Exposure Scale (NES) (Kamitsis and Francis, 2013) have been recognized as valid and reliable measures of nature exposure. These instruments have been adapted and employed in various versions by different research groups.

However, the NCQ is specifically designed to measure nature contact within work contexts, quantifying the frequency and quantity of targeted nature exposures, experiences, and features in a given workweek (Largo-Wight et al., 2011). It assesses three forms of nature contact with a 16-item checklist, offering six numerical response options: outdoor (four items), indoor (six items), and indirect (six items) nature contact. While the NCQ exhibits reasonable test–retest reliability (*r* = 0.85, *p* < 0.01), its internal consistency, as indicated by Cronbach’s α, is somewhat modest (*α* = 0.64). Furthermore, the principal component analysis (PCA) has revealed three factors that roughly correspond to the hypothesized forms of natural contact, yet several items within these factors do not load as expected (Largo-Wight et al., 2011).

In contrast, the NES is a more comprehensive instrument designed to measure exposure to nature as a latent construct, encompassing both direct and sensory contact with natural environments (Francis, 2011, in Kamitsis and Francis, 2013). It captures the frequency and attention dedicated to natural elements or landscapes across diverse contexts, which cannot be effectively encapsulated by a single variable or item (Boateng et al., 2018). While initially created by Francis in 2011 (unpublished research) to assess nature exposure in urban and rural areas across two dimensions–everyday life and outside everyday environments (see Table 1)–the dimensional structure of this scale was not explored at that time. Subsequent studies by Swami and colleagues (Swami et al., 2016) and Wood and colleagues (Wood et al., 2019) tested this scale in different samples, with varying results.

**Table 1:** Inter-item correlation matrix of the nature exposure scale, with means and standard deviations.

	It1-fqQE	It2-atQE	It3-fqNrE	It4-atNrE
It1-fqQE	–	0.492	0.381	0.320
It2-atQE	0.492	–	0.407	0.588
It3-fqNrE	0.381	0.407	–	0.479
It4-atNrE	0.320	0.588	0.479	–
Mean	3.25	3.55	3.33	3.61
SD	1.07	1.00	1.20	1.05

^a^Determinant = 0.349. All rs significant at 0.001 level. SD, Standard Deviation.

Each item on the NES is rated on a 5-point Likert scale (1 = high/a great deal, 5 = low/not much), with higher scores indicating greater exposure to nature. Previous research has indicated acceptable internal consistency (0.70 < α < 0.73; Kamitsis and Francis, 2013; Swami et al., 2016), a one-dimensional factor structure (explaining 54.1% of the variance), and criterion validity for English-speaking adults (Swami et al., 2016). However, a study by Wood and colleagues (Wood et al., 2019) proposed revisions to the NES, eliminating the item assessing exposure to nature in everyday environments (item 1) due to reliability issues. This refinement improved reliability indicators (Cronbach’s *α* = 0.71) and revealed a one-dimensional structure that accounted for 64.0% of the variance. Nonetheless, confirmatory factor analysis (CFA) indicated a suboptimal model fit for both the one-factor, four-item NES model and the initially hypothesized two-factor model, rendering the 3-item version of the original NES untestable.

To enhance both the conceptual robustness and psychometric quality of the NES, Wood and colleagues (Wood et al., 2019) introduced two revised versions of the instrument, incorporating green exercise (GE) and self-reported childhood nature experiences—the NES-II and RNES-II, respectively. NES-II featured two additional items that assessed nature exposure during physical activity, resulting in a five-item scale with a format akin to other items in the NES. RNES-II, similar to NES-II, comprised five items that probed participants’ experiences of nature during childhood, specifically between the ages of 5 and 10.

Both revised versions demonstrated good internal consistency (NES-II α =0.84; RNES-II α =0.89), superior to the original NES (Kamitsis and Francis, 2013), and sound construct validity (NES-II KMO = 0.74; RNES-II KMO = 0.74). Furthermore, they exhibited unidimensionality, as both PCA (explaining 57.4% of the variance for NES-II and 58.5% for RNES-II) and CFA confirmed that overall nature exposure could be reduced to a single factor. However, the fit indices for the one-factor models (with item 1 excluded) were subpar and only improved upon allowing error covariance between items #3 and #5, as suggested by modification indices.

The authors suggested that the versions of the scale without item #1, assessing nature exposure in everyday environments (NES-II and RNES-II), should be labeled the “intentional nature exposure scale” and the “retrospective intentional nature exposure scale,” respectively (Wood et al., 2019). However, the removal of an item from a four-item scale raises concerns about the construct’s validity and its alignment with the underlying theory it aims to measure. Additionally, the acknowledged potential sample bias resulting from online recruitment processes (Wood et al., 2019) underscores the need for further refinement of the NES. Moreover, there is a call for testing the original and newly proposed versions of the instrument in diverse cultural contexts.

In light of these considerations, it becomes imperative to adapt and validate these measures across diverse populations, providing insights into individuals’ subjective experiences, perceptions, and well-being, offering a nuanced understanding that complements objective assessments and contributes to a comprehensive evaluation of the impact of nature on human health. Therefore enabling the comparison of results among different communities and exploring the intricate relationship between exposure to nature and various facets of health–both physical and mental. Consequently, this study’s primary objective is to assess the psychometric properties and theoretical structure of the original NES (Kamitsis and Francis, 2013; Swami et al., 2016; Wood et al., 2019) in the context of Portugal, following its adaptation to the Portuguese language. This involves conducting reliability analyses, as well as exploratory and confirmatory factor analyses, to provide evidence of the scale’s internal consistency and construct, convergent, and divergent validity. The development of validated instruments for measuring exposure to nature in Portuguese-speaking countries will make invaluable contributions to these cultural contexts’ engagement in the scientific discourse.

## 2 Methods

### 2.1 Data collection

For this purpose, data were obtained from two distinct samples: Sample 1, a comprehensive survey on Subjective Well-Being and Health conducted in the Azores Archipelago, Portugal, from 2016 to 2020, and Sample 2, a smaller survey on Gardens and Nature conducted in Terceira Island (Azores) in 2017.

Sample 1 comprised 579 participants, with data from 558 participants used for analysis. These individuals ranged in age from 18 to 87 years (mean age = 33; standard deviation = 14.4) and included both males (*n* = 230; 41.0%) and females (*n* = 327; 59.0%). The participants were evenly distributed between Terceira (42.3%) and São Miguel islands (40.0%), while 6.9% hailed from other Portuguese islands. Educational backgrounds varied, with 48.5% having completed high school, 26.5% having basic education, and the remaining 22.7% holding a university degree. In terms of professions, respondents represented various fields, including commerce (17.3%), health (12.9%), education (7.6%), and public services (6.2%). For the discriminant analysis, a subsample of 11.6% of individuals was selected, with 5.4% having no professional activity explicitly connected to nature exposure. These participants provided demographic information and completed not only the NES but also scales assessing life satisfaction, subjective well-being, connectedness, and relatedness to nature, enabling an assessment of the relationship with NES outcomes.

Sample 2 consisted of 250 participants from Terceira Island, of which 241 completed the NES and provided sociodemographic information. This sample primarily comprised students from lower middle (*n* = 85), upper middle (*n* = 88), and high schools (*n* = 68), with an average age of 13.2 years (standard deviation = 2.2; range: 10–19 years). Gender distribution was nearly equal, with 47.3% being male and 52.7% female. All individuals from both Sample 1 and Sample 2 (totaling n = 799) were included in assessing the psychometric properties of the NES and its dimensionality. However, only individuals from Sample 1 (*n* = 558) were explicitly used to evaluate convergent and divergent validity and discriminative sensitivity.

The original NES by Kamitsis and Francis (2013) was translated into Portuguese by members of the research team who were proficient in English. The translated version was then reviewed and back-translated by individuals fluent in both English and Portuguese to ensure that the original meaning of the items remained intact (Arafat et al., 2016).

Data for Sample 1 were collected by students enrolled in the Health Psychology course of the nursing program at the University of the Azores. These students received specific training provided by A.M.A. and M.L.M. Each student individually administered the survey questionnaire to 10 individuals during the spring of 2016 and 2017, following the receipt of signed informed consent from the participants. It’s important to note that data collection conducted as part of educational courses does not necessitate approval from the Ethics Committee of the University of Azores, and this Committee does not perform post-publication evaluations of the data-gathering process. For Sample 2, data were collected in November 2017 by 12 middle and high school teachers on Terceira Island. These teachers received training from M.dB. and obtained approval from the Ethics Committee of the University of Azores. Given that the sample primarily consisted of adolescents, the teachers also secured written consent from the parents of the students to participate in the study.

All questionnaires were administered anonymously, with strict adherence to personal information confidentiality. Respondents had the option to abstain from answering any question or exit the questionnaire at any point in time.

### 2.2 Data analysis

Statistical analysis was performed using IBM SPSS Statistics version 27 (IBM Corp., Armonk, NY, United States) and the R software (R Core Team, 2019).

#### 2.2.1 Reliability analysis

The internal consistency of the scale was evaluated using Omega coefficient (McDonald, 1999), Cronbach’s alpha tests, including “Alpha” and “Alpha if Item Deleted,” and item-total correlation analysis. McDonald (1999) and Cronbach alpha coefficients must exceed 0.70 (Zinbarg et al., 2005, 2006; Devillis, 2012). Omega coefficient corrects the underestimation bias of Cronbach’s alpha when the assumption of tau equivalence is violated (McDonald, 1999; Graham, 2006). Any items that did not meet these criteria or had a negative impact on reliability were excluded from the analysis.

#### 2.2.2 Principal component analysis

Validity was assessed by examining the relationships between observed and latent variables to identify the emerging construct structure of the original scale. Principal Component Analysis (PCA) was conducted using the principal component condensation method, following the Kaiser rule (Pestana and Gageiro, 2014) in SPSS V.27 to capture and visualize the variance in the data. The sample size ensured a participant-to-item ratio of 140:1, which exceeded the conservative requirement of a 10:1 ratio, thereby reducing the likelihood of sampling errors, in accordance with the principle that “more is always better” (Osborne and Costello, 2004, p.8). As per standard guidelines, all four items underwent PCA because no average correlation with other items fell below 0.40, and the item-total correlation exceeded 0.30.

To assess data factorability, Bartlett’s test of sphericity and the Kaiser-Meyer-Oblim (KMO) measure of sampling adequacy were employed. Bartlett’s test needed to be significant, and the KMO index needed to surpass 0.6 for the data to be considered suitable for PCA.

The determination of the number of factors extracted was based on eigenvalues exceeding 1.0 and examination of the scree plot. Varimax rotation was applied to the input variables to clarify inter-correlations between factors. Factor loadings were interpreted according to Comrey and Lee’s recommendations (Comrey and Lee, 1992), where values >0.71 were considered excellent, >0.63 very good, >0.55 good, >0.45 fair, and > 0.32 poor.

#### 2.2.3 Confirmatory factor analysis

Confirmatory Factor Analysis (CFA) was employed to assess the fit of the NES 1-factor empirical model, which was derived from PCA. This analysis was conducted using the lavaan statistical package (Rosseel, 2012) within R (R Core Team, 2019). To estimate the model, a diagonal weighted least squares mean with variance adjusted robust estimator (DWLS) was utilized. DWLS is particularly suitable for estimating factor loadings in ordinal data, which do not assume a normal distribution, providing less biased and more accurate estimations compared to maximum likelihood estimation (Koğar and Koğar, 2015; Shi and Maydeu-Olivares, 2020).

To evaluate the overall fit of the model using the entire sample, several goodness-of-fit statistics indices were employed. These procedures followed recommendations from Carvalho et al. (2016), Wood et al. (2019), and Schreiber et al. (2006):

- The Tucker-Lewis Index (TLI), also known as the Non-Normed Fit Index (NNFI), assesses how well the tested model improves the fit compared to a null model.
- The Comparative Fit Index (CFI) compares the model’s fit to a nested baseline model that assumes indicator and latent variables.
- The standardized root mean square residual (SRMR) calculates the average difference between predicted and observed variances and covariances in the model, based on standardized residuals.
- The Root Mean Square Error of Approximation (RMSEA) measures the extent to which the models are supported per degree of freedom.

For one-time analyses, the RMSEA, TLI, and CFI indices are preferred (Schreiber et al., 2006). Goodness-of-fit criteria suggest that values of TLI and CFI close to or greater than 0.90 are desirable (therefore used as the cutoff value), while RMSEA and SRMR values should be lower than 0.08.

#### 2.2.4 Convergent and divergent validity and discriminative sensibility analysis

Evidence of convergent and discriminant validity is essential to validate a test and assess the extent to which its scores correlate with other variables as predicted (Furr, 2018). To examine these aspects, Spearman correlation was employed to investigate the relationship between NES scores and measures of connectedness to nature and subjective well-being to assess convergent and divergent validity, respectively.

Convergent associations entail moderate to high correlations, either positive or negative, between the variables being assessed (i.e., instrument scores). These correlations indicate that the variables are related but not identical, suggesting they represent similar yet distinct constructs or latent variables. Conversely, low or non-significant correlations are expected between constructs and latent variables that are unrelated. Nevertheless, variations in these correlations may hint at covariation or predictive relationships, shedding light on the validity of divergent associations. Furthermore, a heterotrait-monotrait (HTMT) correlation ratio was utilized to confirm the distinctiveness of the chosen constructs. HTMT provides enhanced specificity and sensitivity rates (ranging from 97 to 99%). When HTMT values approach 1, it suggests a lack of discriminant validity (Ab Hamid et al., 2017).

To evaluate NES’s ability to differentiate between individuals with varying degrees of nature exposure, a sensitivity and specificity analysis using ROC (receiver operating characteristic) curve analysis was conducted (Tueros and Santos Rosa, 2022). This analysis aimed to determine the potential of NES scores to distinguish between individuals with high and low levels of exposure to nature due to their professional activity provided within the survey demographic information (reported in an open-ended questions).

## 3 Results

### 3.1 Items descriptive statistics and reliability analysis

Table 1 presents descriptive statistics and item correlations for all items within the NES. The highest mean score was observed for item 4, while items 1 and 3, which pertain to participants’ “perceived frequency of exposure to nature,” exhibited lower average scores compared to items 2 and 4, which are associated with the “attention given to nature in the surrounding environment” (Table 1).

Table 2 displays a Cronbach’s alpha and MacDonald’s omega coefficients of 0.76 and 0.77, signifying a commendable level of internal consistency (Zinbarg et al., 2005, 2006; Devillis, 2012). Notably, when items 1 and 3, linked to the perceived “frequency of exposure to nature,” were omitted, Cronbach’s alpha if item deleted increased. These two items, despite having item-total correlations considered good in line with Streiner and Norman (Streiner and Norman, 2008), were found to be relatively weaker in this regard (Table 2). Nevertheless, all values, including Cronbach’s alpha (ranging from *α* = 0.661 to *α* = 0.736), Pearson’s item-total correlations corrected from *r* = 0.487 (*p* < 0.001) to r = 0.636 (*p* < 0.001), and the descriptive statistics (Table 1), consistently demonstrated acceptable or recommended values.

**Table 2:** Correlation and Cronbach alpha if items deleted for nature exposure scale of Kamitsis and Francis (2013).

Items in English (in Portuguese)	Squared multiple correlation	Item-total correlation	α if the item deleted
It1-fqQE–In your everyday home, travel and work environments and activities, please rate your level of exposure to “natural environments” (Por favor, avalie a frequência de exposição a “ambientes naturais” no seu quotidiano doméstico, profissional e em trânsito de uns contextos para os outros).	0.282	0.487	0.736
It2-atQE–How much do you notice the natural environments in your everyday life (Quanto é que repara nos “ambientes naturais” no seu quotidiano)?	0.452	0.636	0.661
It3-fqNrE–Please rate the frequency (how often) of exposure to nature-rich environments outside of your everyday environment (Por favor, avalie a frequência de exposição a “ambientes naturais ricos”).	0.291	0.527	0.721
It4-atNrE–How much notice would you take of the nature in these environments (Quanto é que repara na natureza neste tipo de “ambientes naturais ricos”)?	0.415	0.585	0.684

### 3.2 Principal component analysis

Upon examining the correlation matrix, it became evident that the average correlations of each item with the other two items exceeded 0.40, and the item-total correlations were above 0.30, signifying the suitability of the data for PCA, as shown in Table 2. Furthermore, the respectable Cronbach’s alpha score obtained for the scale (*α* = 0.76) implies that there is no need to eliminate any items from the analysis.

The Kaiser-Meyer-Olkin Measure of Sampling Adequacy (KMO = 0.704) and Bartlett’s test of sphericity [*χ*^2^ (4) = 833.29, *p* < 0.001] indicate that there is substantial common variance for factor analysis, with a reasonable degree of correlation between the variables, allowing the analysis to proceed. Additionally, both the scree plot and the analysis results revealed unidimensionality, as only one eigenvalue exceeded one (2.340), explaining 58.5% of the variance. The factor loadings for the items (Table 3) range from excellent (items 2, 3, and 4) to very good (item 1).

**Table 3:** Factor loadings for NES (4-item scale).

	Factor loading
It1-fqQE	0.701
It2-atQE	0.827
It3-fqNrE	0.733
It4-atNrE	0.792

### 3.3 Confirmatory factor analysis

**Figure 1:**
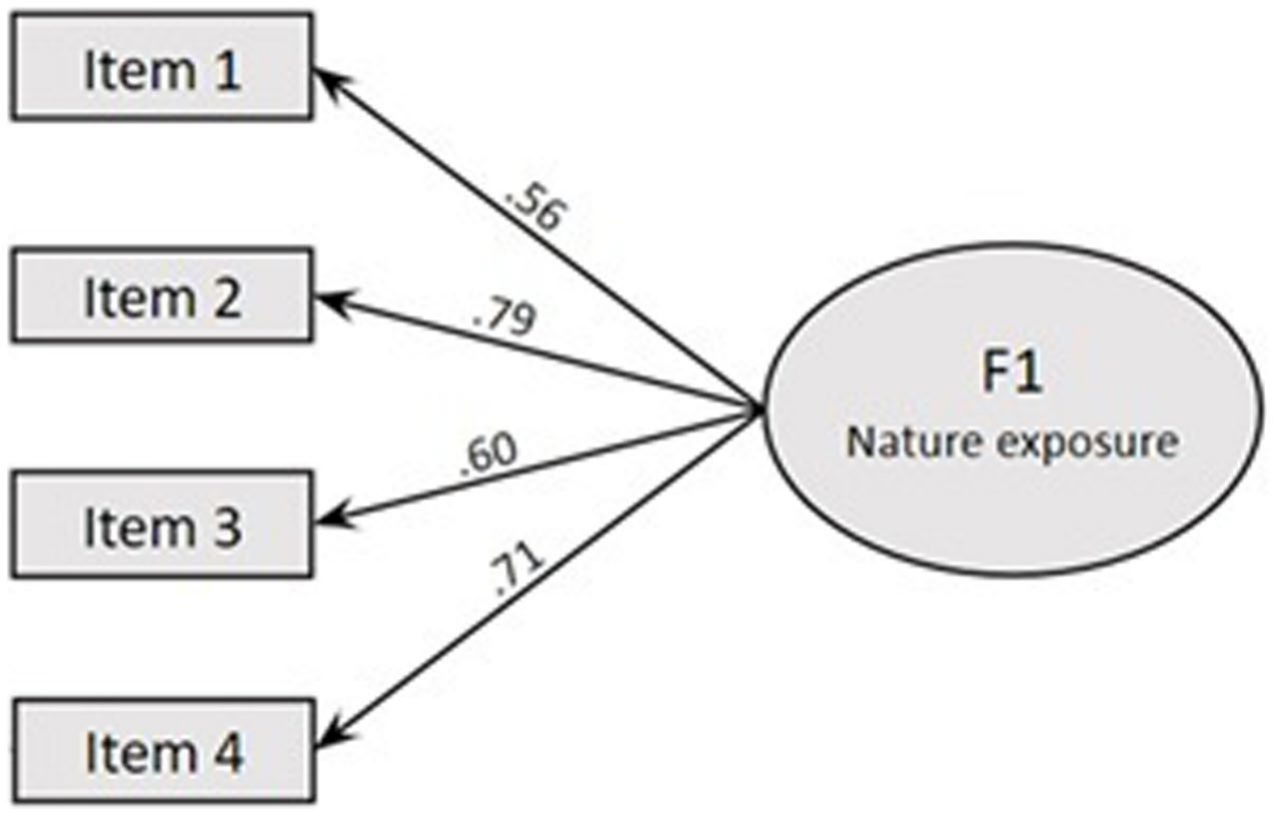
Fit for the one-factor four-item model of NES (taken from PCA).

The CFA was performed to scrutinize the structural model, evaluating the goodness of fit for the one-factor empirical model derived from PCA, as illustrated in Figure 1, which assesses overall nature exposure.

The robust fit indices yielded the following results: CFI of 0.986 (CFI ⋝ 0.95), TLI of 0.957 TLI ⋝ 0.90, RMSEA of 0.083 (with a 90% confidence interval ranging from 0.061 to 0.107; RMSEA ⋜ 0.08), and SRMR of 0.043 (SRMR ⋜ 0.08). These values collectively indicate a very good fit adjustment, except for the RMSEA value deemed acceptable (Figure 1).

### 3.4 Convergent and divergent validity and discriminative sensibility analysis

Spearman’s correlation analysis unveiled a noteworthy positive association between scores on the original 4-item NES and the brief Nature Relatedness measure (NR-6; Nisbet et al., 2009; rho = 0.558, *p* < 0.001), as well as the 14-item Connectedness to Nature scale (CNS; Mayer and Frantz, 2004; rho = 0.400, *p* < 0.001; Table 4). These findings substantiate our predictions, which were based on evidence and theory, indicating that the moderate convergent links between exposure to nature and connectedness to nature imply that these variables are related yet distinct.

**Table 4:** Relationship between NES scores, well-being, and connectedness to nature variables (Spearman Correlation coefficients).

		Mean	SD	4-item NES	
				rho value	Value of *p*
Connectedness to nature	CNS	45.41	9.371	0.400*	0.000
	NR-6	20.08	5.215	0.558*	0.000
Subjective well being	SH	17.59	3.280	0.108*	0.009
	SWLS	17.15	3.763	−0.010	0.814

^*^Correlation is significant at the 0.01 level (2-tailed).

Conversely, the NES demonstrated very weak or almost negligible correlations with two subjective well-being instruments (as shown in Table 4): Subjective Happiness (SH, Lyubomirsky and Lepper, 1999; rho = 0.108, *p* < 0.001), and the Satisfaction with Life Scale (SWLS; Diener et al., 1985; rho = −0.010, *p* > 0.05). Furthermore, the divergent validity was confirmed by HTMT values of 0.16 for NES – SH and 0.07 for NES – SWLS.

**Figure 2:**
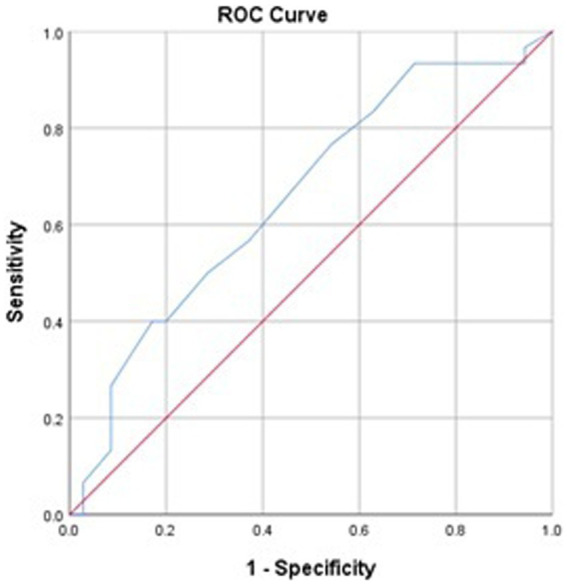
Sensitivity and specificity of NES (ROC curve) to evaluate the discriminative capability within professionals with higher (*n* = 31) and lower (*n* = 35) exposure to nature.

The NES exhibited the ability to differentiate between individuals engaged in professional activities with higher (*n* = 31) and lower (*n* = 35) exposure to nature. It displayed a statistically significant ROC curve (Figure 2) with a low AUC value of 0.658 (with a minimum suggested AUC of 0.70) and a standard error (SE) of 0.68, *p* < 0.05 (95% CI 0.525–0.792). This indicates that NES scores can be used to distinguish individuals at random.

These results were further supported by the equality test, which revealed significant distinctions between the groups [*χ*^2^ (1) = 4.052, *p* < 0.05] and a Wilks’ lambda value of 0.94, indicating low yet significant discriminative ability.

## 4 Discussion

This study intends to adapt and validate the NES measure by assessing the psychometric properties and theoretical structure of the original NES (Kamitsis and Francis, 2013; Swami et al., 2016; Wood et al., 2019) in the portuguese context. Therefore enabling the comparison of results among different cultural communities and exploring the relationship between exposure to nature and various facets of behavior and health–both physical and mental.

The health benefits associated with exposure to natural environments have been extensively substantiated in the existing literature, bolstered by numerous empirical studies (Diener et al., 1985; Largo-Wight et al., 2011; Keniger et al., 2013; Russell et al., 2013; Korpela et al., 2017; Hyvönen et al., 2018; Repke et al., 2018; Jo et al., 2019; Trøstrup et al., 2019; Wyles et al., 2019; Jimenez et al., 2021). As such, it has become increasingly imperative to devise methods for assessing exposure to nature, particularly in light of the disparities observed across various studies (Trøstrup et al., 2019; Holland et al., 2021).

**Figure 3:**
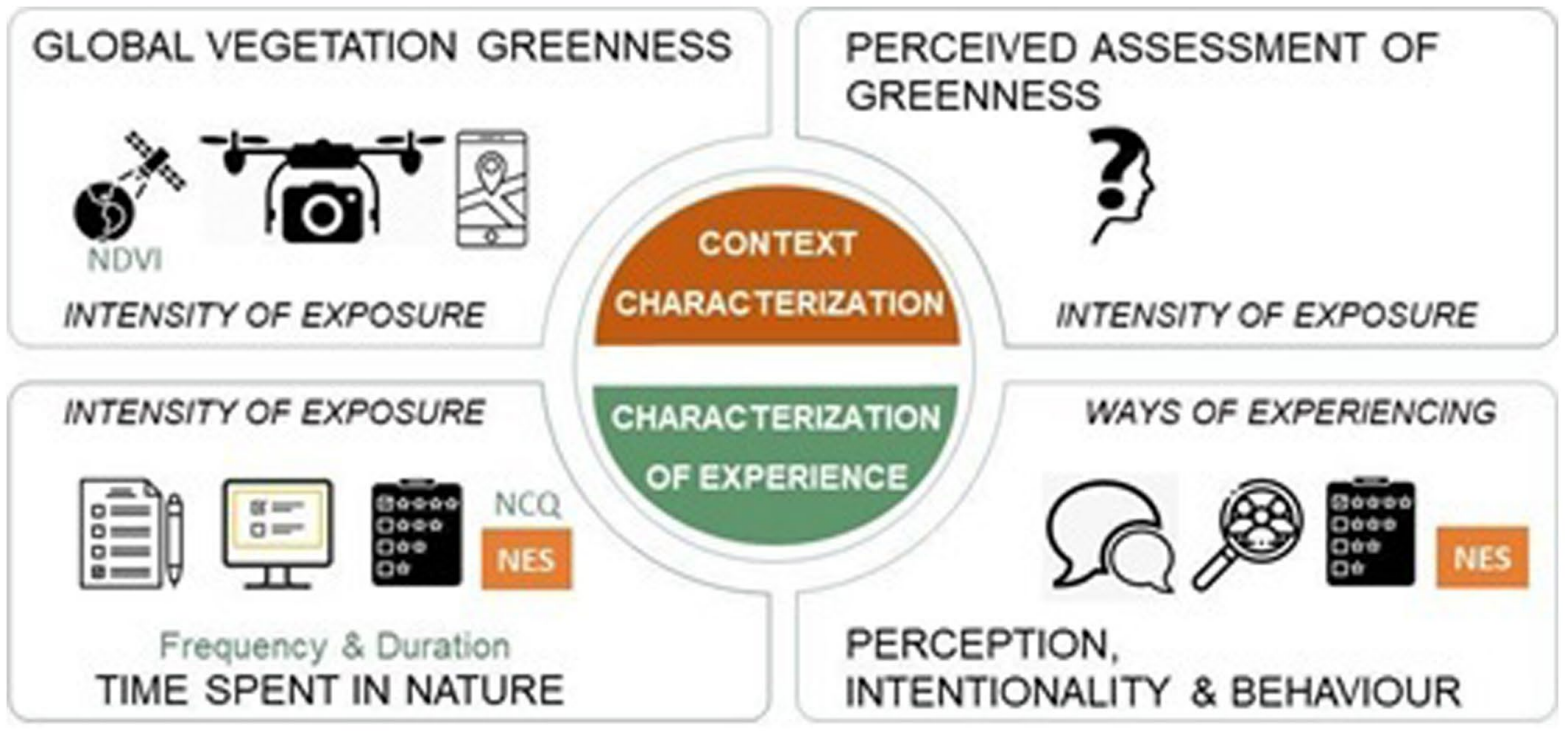
Indicators assessed in different nature exposure metrics.

Many studies have aimed to assess exposure to nature by employing indices, remote sensing technologies, or global positioning systems to estimate the extent of exposure to greenery (Holland et al., 2021). However, these approaches are not without their technical and conceptual limitations (White et al., 2017; Giannico et al., 2022) since they do not evaluate the actual experience of nature. Moreover, these studies face the challenge of cross-country comparability due to cultural differences in how nature is perceived and understood (Holland et al., 2021). Consequently, several scholars have emphasized the need to shift from context characterization to experience characterization to achieve more accurate assessments (see Figure 3). Self-reported measures enable researchers to consider cultural and contextual variations in individuals’ experiences with nature, especially in longitudinal studies aiming to track changes in individuals’ contact with nature over time, regardless of changes in the natural contexts experienced (Thompson and Aspinall, 2011). Other reasons underscore the relevance of self-reported measures in assessing exposure to nature, beyond the insights provided into the qualitative aspects of people’s interactions with nature. For instance, they allow participants to reflect on the meaning of their nature encounters, offering insights into how nature fits into their overall life narrative. Additionally, self-reported measures provide a cost-effective and practical means of data collection, enabling researchers to gather information on individuals’ exposure to nature on a large scale. This is especially important for studies aiming to explore associations between nature exposure and various outcomes across diverse populations (Largo-Wight et al., 2011; White et al., 2019; Soga et al., 2021).

However, the more relevant aspect appears to be the fact that self-reported measures allow for a comprehensive assessment of exposure to nature beyond mere quantitative metrics. They can encompass the frequency, duration, and quality of nature experiences, providing a holistic understanding of how individuals engage with natural environments. This includes assessing behavioral patterns in nature, such as outdoor activities, preferences for specific natural settings, and motivations and intentions for future interactions with nature. Capturing individual differences helps tailor interventions or policies promoting nature engagement and positive impacts. This nuanced information is valuable for uncovering the intricacies of the relationship between nature exposure and well-being (Largo-Wight et al., 2011; Shanahan et al., 2016a; Cox et al., 2017; Rugel et al., 2017; Holland et al., 2021). By analyzing a self-report measure of exposure to nature, this work contributes to advancing the literature by addressing the limitations of alternative research methods. It adds psychometric evidence that helps clarify previous results and broadens the universe of research to new cultural contexts. Presently, the NES stands out as the sole psychometric tool and self-report method capable of characterizing both the frequency and the quality of nature exposure, encompassing everyday natural settings unintentionally frequented and more deliberate visits to enriched natural environments, offering a more comprehensive evaluation. The NES is uniquely positioned to evaluate the extent to which natural surroundings impact human well-being and to furnish precise data on this matter, setting it apart from other published works (e.g., Kamitsis and Francis, 2013; Wood et al., 2019; Baceviciene et al., 2021). This underscores the importance of conducting further validation in diverse cultural contexts.

Despite the NES’s initial introduction in 2013 (Kamitsis and Francis, 2013), its psychometric validation was only undertaken by Swami and colleagues in 2016 (Swami et al., 2016), and subsequently by Wood and colleagues in 2019 (Wood et al., 2019). Notably, these two studies yielded distinct results, both highlighting the need for additional research.

The findings pertaining to the NES’s reliability in the Portuguese cultural context revealed a reasonably consistent instrument, as evidenced by a Cronbach’s alpha of 0.76 and McDonald Omega of 0.77. This Cronbach’s alpha score slightly surpassed values reported by Kamitsis and Francis (2013), *α* = 0.73; Swami et al. (2016); *α* = 0.70 for women and *α* = 0.72 for men, and Wood et al. (2019); *α* = 0.69 for the 4-item NES and *α* = 0.71 for the 3-item NES. These values collectively indicate an acceptable or satisfactory level of internal consistency (Cronbach alpha and McDonald Omega >0.70) for the scale.

PCA conducted on the four NES items for the entire sample unveiled a sufficient degree of common variance. This was corroborated by the Kaiser–Meyer–Olkin (KMO) measure of sampling adequacy (KMO = 0.704) and Bartlett’s test of sphericity [*χ*^2^ (4) = 833.29, *p* < 0.001], which indicated that the correlation matrix was suitable for factor analysis. Moreover, an eigenvalue exceeding one (2.340) suggested that the unidimensional solution explained 58.5% of the variance. Notably, all items exhibited robust factor loadings, with even the lowest loading (0.701 for item 1) indicating that the factor extracted sufficient variance from that particular variable. These findings closely align with those reported in studies involving English-speaking adults by Swami et al. (2016) and the more recent findings by Swami et al. (2022). In contrast, they diverge from the results obtained by Wood et al. (2019), where the cutoff values from correlation inspection matrices (0.40) necessitated the exclusion of item 1 (0.264). However, such exclusion would compromise the scale’s conceptual integrity, as it assesses the frequency of exposure to everyday environments.

Furthermore, CFA of the NES’s one-factor model, as illustrated in Figure 1, confirmed the appropriateness of the unidimensional model, with the model displaying good fit indices. This is an unambiguous contrast to the adjustment difficulties encountered by Wood et al. (2019) with the original NES 4 and 3-item empirical versions. Notably, none of the eight models tested by the authors, including 5 or 6-item modified versions of NES (Wood et al., 2019), such as the “green exercise” experience (NES-II) and the retrospective child experience of nature exposure (RNES-II), met the criteria for goodness of fit statistics. Only after employing modification indices to enhance model fit did the 5-item versions of NES-II and RNES-II models achieve an adequate fit.

The correlations between the NES and two other nature-related scales that assess nature connectedness, CNS (Mayer and Frantz, 2004), and NR-6 (Nisbet et al., 2009), demonstrated moderate yet significant associations. This substantiates the conceptual affinity between these constructs or latent variables, although they are not identical. This aligns with findings reported by Kamitsis and Francis (2013), Swami and colleagues (Swami et al., 2016), and Wood and colleagues (Wood et al., 2019) concerning convergent validity with modified NES (NES-II and RNES-II).

Conversely, the NES exhibited notably weak and almost non-existent correlations with two measures of subjective well-being, SH (Lyubomirsky and Lepper, 1999), and SWLS (Diener et al., 1985). This further supports the adequate divergent validity of the NES, highlighting the distinctiveness of these constructs or latent variables. Indications of divergent validity for the NES were also observed in other studies exploring mental health variables (depression, anxiety, stress, and psychological well-being) conducted by Wood and colleagues (Wood et al., 2019), as well as self-esteem by Swami and colleagues (Swami et al., 2016).

In accordance with Farrell and Rudd (2009), establishing the discriminative capability of an instrument necessitates more than just reliability and validity indices. The NES’s sensitivity and specificity analysis, performed using the ROC curve (Tueros and Santos Rosa, 2022), corroborated its capacity to distinguish between groups of participants engaged in professional activities expected to entail varying degrees of exposure to nature.

In summary, the original 4-item NES scale has proven to be a robust instrument for assessing both intentional and unintentional exposure to nature, encompassing rich and everyday settings alike. Its adaptation and validation in the Portuguese context fill a critical gap in the literature, providing a culturally sensitive tool for assessing nature exposure, enabling future cross-cultural research (Jimenez et al., 2021). Additionally, this not only contributes to the cross-cultural applicability of the scale but also facilitates future research on the NES in Portuguese-speaking regions (Figure 2).

## 5 Conclusion

The significance of contact with natural environments for human health and overall well-being is well-documented; however, assessing this contact has often been limited to estimating the level of surrounding greenery or quantifying time spent in nature. The NES stands out as one of the few instruments designed not only to gage the intensity of exposure but also to delve into the nuances of how individuals experience nature, including the degree of attention they pay to it, whether in enriched natural settings or everyday environments.

In light of our research findings, the original version of NES emerges as a concise and easily administered paper-and-pencil tool for evaluating nature exposure, a perspective also corroborated by Swami and colleagues (Swami et al., 2016). This contrasts with the challenges in model fit experienced in the analysis conducted by Wood and colleagues (Wood et al., 2019). The divergence in results may underscore recurring challenges in interpreting model fit and defining appropriate cutoff values, as suggested by Hu and Bentler (1999) and Perry et al. (2015).

The robust psychometric properties of NES facilitate the replication of studies on the benefits of nature exposure in various cultural contexts, extending beyond Anglo-Saxon regions. This opens up opportunities to investigate whether the impact of nature exposure is culture-neutral or culture-dependent, exploring its variations across different life stages. Additionally, there are promising avenues for research into how nature exposure influences individuals’ connectedness to and commitment to the protection and conservation of nature. NES could also play a valuable role in formal and informal learning contexts, particularly in Science, Citizenship, and Environmental Education (Silva et al., 2023).

Nonetheless, it’s crucial to emphasize that the convenience sampling method employed in this study warrants a degree of caution when it comes to making broad generalizations based on the results, since the sample could not be representative of the Portuguese population. Subsequent research endeavors using more rigorous stratified or randomized sampling techniques should be undertaken to validate these findings.

Furthermore, the present study relied on data from a survey conducted in the Azores (Portugal) to explore whether individuals’ recalled sense of connectedness to nature varies in relation to the type and quality of the natural environments they frequented. This investigation also aimed to discern how such variations are associated with their recollected experiences of psychological restoration. The study’s outcomes underscore the fact that both the degree of connectedness to nature and the recollection of psychological restoration are notably influenced by the specific attributes of the natural environment visited.

Specifically, rural green and coastal settings, particularly those recognized for their superior quality, such as protected or designated areas, were consistently linked with more vivid recollections of connectedness to nature and heightened feelings of psychological restoration. Collectively, these findings enrich the expanding body of literature focused on the psychological advantages afforded by visits to natural settings. Additionally, they hold the potential to inform policy decisions and guide environmental management practices by offering insights into the prioritization of safeguarding and ensuring accessibility to natural sites that confer these significant benefits.

The emphasis on self-reported measures in this study enhances our understanding of the subjective dimensions of nature exposure, and the use of the NES contributes to the advancement of the literature by offering a robust, culturally validated tool for assessing nature exposure and its impact on well-being.

## Data availability statement

The raw data supporting the conclusions of this article will be made available by the authors, without undue reservation.

## Ethics statement

The studies involving humans were approved by the University of Azores ethics committee. The studies were conducted in accordance with the local legislation and institutional requirements. Written informed consent for participation in this study was provided by the participants' legal guardians/next of kin.

## Author contributions

AP: Conceptualization, Data curation, Formal analysis, Methodology, Software, Validation, Writing – original draft, Writing – review & editing. AA: Conceptualization, Formal analysis, Funding acquisition, Investigation, Methodology, Project administration, Resources, Supervision, Validation, Visualization, Writing – original draft, Writing – review & editing. NT: Formal analysis, Writing – review & editing. AS: Investigation, Resources, Writing – review & editing. EP: Data curation, Investigation, Resources, Writing – review & editing. MB: Investigation, Resources, Writing – review & editing. IA: Investigation, Resources, Writing – review & editing. ML: Investigation, Resources, Writing – review & editing. RG: Data curation, Funding acquisition, Investigation, Resources, Writing – review & editing.
